# Genetic and environmental determinants of insect herbivore community structure in a *Betula pendula *population

**DOI:** 10.12688/f1000research.3-34.v1

**Published:** 2014-01-31

**Authors:** Tarja Silfver, Matti Rousi, Elina Oksanen, Heikki Roininen

**Affiliations:** 1 Faculty of Science and Forestry, Department of Biology, University of Eastern Finland, FIN-80101 Joensuu, Finland; 2 Vantaa Research Unit, Finnish Forest Research Institute, FIN-01301 Vantaa, Finland

## Abstract

A number of recent studies have shown that intraspecific genetic variation of plants may have a profound effect on the herbivorous communities which depend on them. However less is known about the relative importance of intraspecific variation compared to other ecological factors, for example environmental variation or the effects of herbivore damage. We randomly selected 22 *Betula pendula *genotypes from a local population (< 0.9 ha), cloned them and planted cloned seedlings on two study sites separated at a regional scale (distance between sites about 30 km) to examine an insect community of 23-27 species on these genotypes. *B. pendula* genotypes did not differ in their species richness, but the total mean abundance and the structure of the insect herbivore community was significantly affected by the genotype, which could account for up to 27% of the total variation in community structure. *B. pendula* genotype accounted for two to four times more variation in the arthropod community structure than did environmental (block) variation on a local scale, while on a regional scale, genotypic and environmental (site) variation accounted for 4-14% of the arthropod community structure. The genetic effects were modified by environmental variation on both a local and regional scale over one study year, and locally, the largest part of the variation (38%) could be explained by the genotype × environment (block) interactions. Suppression of insect herbivores during one growing season led to changed arthropod community structure in the following growing season, but this effect was minimal and could explain only 4% of the total variation in insect community structure. Our results suggest that both genetic and environmental factors are important determinants of the community structure of herbivorous insects. Together these mechanisms appear to maintain the high diversity of insects in *B. pendula *forest ecosystems.

## Introduction

Genetic variation within one species can affect the structure and dynamics of associated communities and entire ecosystems ^1, 2^. This may be considerable, especially for keystone species, such as forest trees, which serve as food and habitat for numerous primary consumers. A vast number of studies have already shown that arthropod communities respond to genetic differences among individual plants within interspecific hybridizing complexes (e.g. *Eucalyptus* ^3^, *Salix* ^4^, *Populus* ^5^, *Quercus* ^6^) or specific genotypes within species (e.g. *Oenothera biennis* ^7^, *Eucalyptus globulus* ^8^, *Solidago altissima* ^9^, *Populus angustifolia* ^10^). However, it has recently been argued that the role of plant genetic variation in structuring arthropod communities has been considerably inflated due to the common methodological flaw that genotypes are collected from diverse and often distant environments, which maximizes genetic variation, whilst experiments are performed in a single common garden where environmental variation is minimized ^11, 12^. Indeed, when this mismatch in scale was avoided in the experimental design, spatial processes relegated host plant genotype to a secondary role in structuring insect communities of *Quercus robur* L. ^13^. Whether this applies to all systems is, however, not yet known.

Genes encounter a range of environments in nature and it has long been recognized that genetic determination of plant susceptibility to a herbivorous insect depends on environmental context ^14^. However, most studies that have examined the role of genotype × environment interactions in the abundance and distribution of herbivorous species, have used only one or a few closely related herbivore species (e.g. ^15– 18^), and much fewer studies have examined genotype × environment interactions in a community context ^7, 13, 19, 20^. It is well recognized that we know too little of the relative importance of intraspecific genetic variation compared to other ecological factors that also influence multi-trophic communities and ecosystem processes ^11^. Thus, the examination of genotype × environment interactions in a community context may be essential for improving our knowledge in the developing field of community genetics.

Silver birch ( *Betula pendula* Roth) is an ideal tree species in which to examine the mechanisms of plant-herbivore interactions and the community-level consequences of trait variation, because the species shows remarkable genetic variation in its resistance to herbivores ^21– 24^. In addition, the genetic variation of secondary metabolites ^26^, nutrient concentrations ^27^, and phenological traits ^28, 29^ of *B. pendula* are known to be substantial, and all these traits are known to affect herbivores and higher trophic level interactions ^2, 30, 31^. Most of the studies that have been conducted using *B. pendula* have used genotypes that were originally randomly selected from a local *B. pendula* population, i.e. from a naturally regenerated forest stand < 0.9 ha. None of these earlier studies have, however, investigated the within-population genotypic variation in *B. pendula* insect herbivore species richness and community composition. We cloned 22 *B. pendula* genotypes, planted them in two common gardens separated at a regional scale (distance between sites about 30 km), and studied the relative importance of genetic variation in community patterns, comparing both local and regional environmental variation. In addition, we examined how strongly herbivores themselves can modify arthropod communities associated with *B. pendula* by suppressing herbivores from half of the saplings over one growing season in one common garden and surveying their arthropod communities the following season.

## Materials and methods

### Plant material and study sites

The 22 different genotypes of *B. pendula* were cloned during spring 1998 from randomly selected *B. pendula* trees taken from a naturally regenerated *B. pendula* - *B. pubescens* Ehr forest in Punkaharju, southeastern Finland (61°48′ N, 29°18′ E), to study genetic variation in phenology, growth, reproduction and resistance-related traits among individual birch trees ^25^. Sampling was stratified random sampling: six spots where forest lift could be transferred were first selected around the forest, and 2–5 trees within the reach of forest lift in each spot were then randomly (by throwing a coin) selected for our study purposes. *B. pendula* is predominantly a sexual species, but genotypes can be cloned for study purposes or for plantations using standard tissue-culture methods ^32^. Cloned *B. pendula* saplings were planted at the growing sites (i.e. common gardens, each approximately 0.25 ha) in June 1999 to find out the degree to which the genotype and environment affect birch traits and to test how genotypes differ in their response to the environment ^26^. The Kuikanniitty study site (61°47′ N, 29°21′ E) is an abandoned cultivated field and the Parikkala study site (61°36′ N, 29°36′ E) is Myrtillus type forest ^33^. Soil type was defined as fine sandy till for both sites ^26^. The distance between these sites was around 30 km and they were situated at approximately the same altitude (Kuikanniitty 79 m and Parikkala 93 m above sea level). Thus, the mean summer (June–August) temperatures were very similar at these sites: in 2002 mean temperatures were 17.6°C and 17.9°C and in 2003 they were 15.9°C and 15.6°C in Kuikanniitty and Parikkala, respectively. Both study sites were divided into six blocks, each of which included four saplings from each genotype. To prevent edge effects, the experimental saplings were surrounded by one row of extra saplings. From each block, one of the four saplings of a total of 22 genotypes was randomly selected for the present study in order to have six replicates per genotype.

In addition, we collected additional data from Kuikanniitty in 2003 to investigate the effect of previous insect herbivory on insect community structure and abundance, and surveyed one extra sapling from each block and genotype. These extra saplings were protected from insect herbivory in the previous growing season by regular sprayings with synthetic pyrethrin ^23^, which has no direct or side effects on the growth or chemistry of birch seedlings ^34^.

### Measuring insect abundance and species richness

The insect herbivore community of each sapling was assessed by surveying the abundance of 23 (in Parikkala 2002) or 27 (in Kuikanniitty 2002–2003, and Parikkala 2003) insect taxa from diverse orders (Lepidoptera, Hymenoptera, Coleoptera, Diptera, Hemiptera; Table 1). These taxa were generally the most abundant taxa in both sites. However, species that were rare in both sites were included in the surveys as well. Species identifications were undertaken following Saalas ^35^ species identification guide, using several web pages ( http://www.funet.fi/pub/sci/bio/life/insecta/index.html; http://www.leafmines.co.uk/index.htm; http://www.bladmineerders.nl/; http://www.nrm.se/) with the assistance of specialists. *Euceraphis betulae* eggs were counted from the side of twelve (2002) or eight (2003) topmost buds in April before budburst. However, in 2002 *E. betulae* eggs were counted from two saplings per genotype per block (sum of the eggs on the sides of 24 buds was used in the analysis), because regular sprayings with synthetic pyrethrin on the other sapling was started only after egg counts in both sites. *Trichiosoma* sp. pupae were counted in April/May when the timing of budburst of the same saplings was observed (Possen, submitted manuscript). The abundance of *Eriocrania* sp. was determined at the end of June, *Deporaus betulae* at the beginning of July and Heteropteran 1 (sap sucker) in August. *Croesus septentrionalis* larval colonies and the number of larvae in each colony were recorded along with *Eriocrania* and *D. betulae* measurements in both years. The abundance of all other insects were determined indirectly by counting damaged leaves at the beginning of September in both years, since the damage caused by most of the surveyed taxa remained identifiable for a long time after the initial damage.

**Table 1.** Description of the 27 taxa surveyed for their abundance among 22 genotypes in Kuikanniitty and Parikkala field experiments 2002 and 2003.

		2002		2003	
		Kuikanniitty	Parikkala	Kuikanniitty	Parikkala
Taxa	Identification	Total number of insects/damage counted			
Lepidopteran miners/rollers					
Gracillaridae 1 (miner)	*Phyllonorycter cavella*	282	123	53	34
Gracillaridae 2 (miner)	*Phyllonorycter* sp. 1	61	26	12	4
Gracillaridae 3 (miner)	*Phyllonorycter* sp. 2	19	7	3	3
Gracillaridae 4 (miner)	*Parornix betulae*	114	42	40	10
Gracillaridae 5 (miner)	*Parornix* sp.	30	11	20	0
Eriocranidae (miner)	*Eriocrania* sp.	536	2007	746	2374
Pyralidae (roller or tier)	tentatively *Euzophora fuliginosella*	67	77	135	142
Tortricidae (galler)	*Epinotia tetraquetrana* ^a^	159	136	159	136
Nepticulidae (miner)	*Stigmella* sp. 1	40	53	7	1
Incurvanidae (miner)	*Phylloporia bistrigella*	125	6	30	8
Geometridae (roller or tier)	*Rheumaptera hastata*	11	6	4	0
Gelechiidae (roller or tier)	tentatively *Teleiodes* sp.	87	-	211	37
Mircolepidoptera 1 (roller or tier)		64	65	188	60
Lepidoptera 1 (roller or tier)		8	2	3	0
Lepidoptera 2 (miner)		12	-	7	3
Lepidoptera 3 (roller or tier)		0	1	13	1
Lepidoptera 4 (miner)		142	7	152	82
Coleopterans					
Attelabidae (roller)	*Deporaus betulae*	62	14	157	133
Curculionidae (miner)	*Orchestes rusci*	54	127	23	12
Hymenopterans					
Tenthredinidae 1 (miner)	tentatively *Fenusa pumila*	149	109	66	59
Tenthredinidae 2 (leaf feeder)	*Hemichroa australis*	167	-	52	18
Tenthredinidae 3 (leaf feeder)	*Croesus septentrionalis*	108	7	34	0
Cimbicidae (leaf feeder)	*Trichiosoma* sp.	6	2	2	0
Dipterans					
Agromyzidae (miner) 1	*Agromyza alnibetulae*	24	11	33	6
Cecidomyiidae (miner) 1		0	0	1	19
Hemipteran					
Aphidoidea (sap sucker)	*Euceraphis betulae*	996	2640	40	114
Heteropteran					
Heteropteran 1 (sap sucker)		92	-	284	466

^a^ *E. tetraquetrana* counts represent the damage during the whole lifetime of the saplings (see Materials and methods). Note also that years are not directly comparable because of the changed sampling protocol between years.

In general, the insect abundance in 2002 was determined by surveying the whole sapling. The mean height of these saplings at the end of 2002 was 253 ± 4.3 cm (mean ± SE) in Kuikanniitty and 227 ± 3.8 cm in Parikkala. Because *B. pendula* genotypes differ in their height and diameter growth ^23^ and large saplings may harbor more insects than smaller saplings, we determined the whole sapling “surface area” and used it as a covariate hereafter called “size index” in statistical analysis. Surface area was determined by photographing each sapling sideways from their southern side against a white background, converting the picture to a black and white silhouette picture in Adobe Photoshop 7.0 and determining the number of black pixels (i.e. leaf and branch area) within the picture. The number of pixels was converted to m ^2^ using the number of pixels of a known area as a reference. The amount of pixels significantly (p < 0.001) explained over 73% of the sapling volume [Y = (3.14 * {base diameter/2} ^2^ * height)/3] in both sites. The abundance of *Phyllonorycter cavella*, *Phyllonorycter* sp. 1, *Parornix betulae* and *Parornix* sp. was not examined on the whole sapling, but was determined as the damage (i.e. number of mines per each species) found within a period of 30 seconds. The period of time (30 sec) was chosen so that even the smallest saplings had leaves uncounted when the time was up.

Since the method of assessing herbivore abundance/resistance by time counts has been successfully used in the past ^35, 36^ we decided to use time counts to determine the abundance of almost all taxa (except *E. betulae*, *Trichiosoma* sp. and *C. septentrionalis*) in 2003. The same person undertook all surveys. The abundance of easily visible damage (large mines and rolls) of *Eriocrania* sp., *D. betulae* and Heteropteran 1 were determined as the number of damaged areas found within a period of 30 seconds. *Epinotia tetraquetrana* “knobs” in the branches of the saplings were counted within a period of 20 seconds in 2003 starting at the top of the tree. Since the “knobs” in the branches remain visible for years and we did not separate different year’s growth while surveying, the values represent the accumulation of *E. tetraquetrana* damage during the last few years. Therefore the same values were used in both years’ insect community analyses. The abundance of all other 20 taxa in 2003 was determined within a single time count of each sapling at the beginning of September. To examine a similar proportion of each sapling, they were divided into three size categories according to their height and number of leaves. Small saplings (average height 2.8 and 3.2 m in Parikkala and Kuikanniitty, respectively) were surveyed for 30 seconds, average sized saplings (3.5 and 3.9 m in Parikkala and Kuikanniitty, respectively) for 60 seconds and large saplings (4.5 m in Kuikanniitty, large saplings were not found in Parikkala) for 120 seconds. Surveying time was used as a covariate called size index in statistical analysis.

### Data analyses

All multivariate analyses were performed with Primer 6 (Primer-E Ltd, United Kingdom). The full data matrix consists of the abundance of 23–27 (23 in Parikkala 2002) insect species in 264 saplings (22 genotypes, 6 blocks, 2 sites) that were surveyed in two consecutive years. All surveyed insect species were included in the statistical analysis when sites were tested separately, but those four species that were not surveyed in Parikkala 2002 were excluded also from Kuikanniitty 2002 data when sites were compared. Arthropod community composition data was analyzed using non-parametric multivariate analysis of variance (PERMANOVA), which is well suited to non-normal ecological data such as ours ^38, 39^. Years were analyzed separately in all statistical tests, because of the changed sampling protocol between years (surveying the whole tree in 2002, using time counts in 2003). All data was fourth root transformed prior to analysis to reduce differences between common and rare species. The semimetric Bay-Curtis distance, which generally seems to provide the most meaningful measure of dissimilarity in ecological community structure ^39^, was used to calculate distances between each pair of observations. The resulting distance matrix was used to obtain p-values using a random subset of 4999 permutations in PERMANOVA. The permutation method was permutation of residuals under a reduced model. The statistical model was designed to test the effect of genotype, site, block (nested within site) and the interaction of genotype × site using sapling size index (sapling surface area in 2002 and surveying time in 2003, see above) as a covariate. Site was treated as a fixed factor and block and genotype as random factors in the model. In addition to these analyses, we separately tested the effect of genotype and block on insect assemblages in each site and year to calculate the proportion of variance explained by *B. pendula* genotype and local environment (i.e. replicated block). Additional data collected from those saplings that were protected from insect herbivory in the previous growing season in Kuikanniitty 2003, were combined with the Kuikanniitty 2003 non-treated sapling data prior to analyzing the effects of insect removal, block and genotype, and their two-way interactions with the insect assemblages with PERMANOVA. Sapling size index was used as a covariate.

To visualize the multivariate patterns among observations, non-metric multidimensional scaling (nMDS) was performed on the Bay-Curtis distances. The distance among centroids for groups of samples was determined prior to nMDS to increase clarity, e.g. when the whole data was visualized we had 88 genotype-site-year points (22 genotypes in 2 sites over 2 years) instead of 528 genotype-block-site-year points. To visualize the effect of genotype in individual site and year, we separately determined the distance among genotype centroids in each site and year and produced one nMDS plot from each of these “environments”. Additional Kuikanniitty 2003 data combined with Kuikanniitty 2003 raw data was used to visualize the effect of insect removal on insect assemblages using nMDS on the genotype centroids of those saplings that were either protected from herbivory or grown under natural herbivory.

Species richness (number of species/sapling) and total mean abundance (number of herbivores/sapling) was statistically tested by analysis of covariance using SPSS 20.0.0.1 (IBM SPSS Statistics) General Linear Models (GLM) procedure. Those four species that were not surveyed in Parikkala 2002 were excluded also from Kuikanniitty species richness and total mean abundance calculations to better enable site comparisons. Genotype and block (nested within site) were treated as random factors and site as a fixed factor in the statistical model while sapling size index was used as a covariate. Additional Kuikanniitty 2003 data combined with Kuikanniitty 2003 basic data was used to analyze the effects of insect removal, block and genotype, and their interactions with the species richness and total mean abundance. Genotype and block were treated as random factors and insect removal as a fixed factor while sapling size index was used as a covariate. Total mean abundance was log(x+1)-transformed to equalize the error variances across groups in both analyses.

## Results

**Figure 1.**
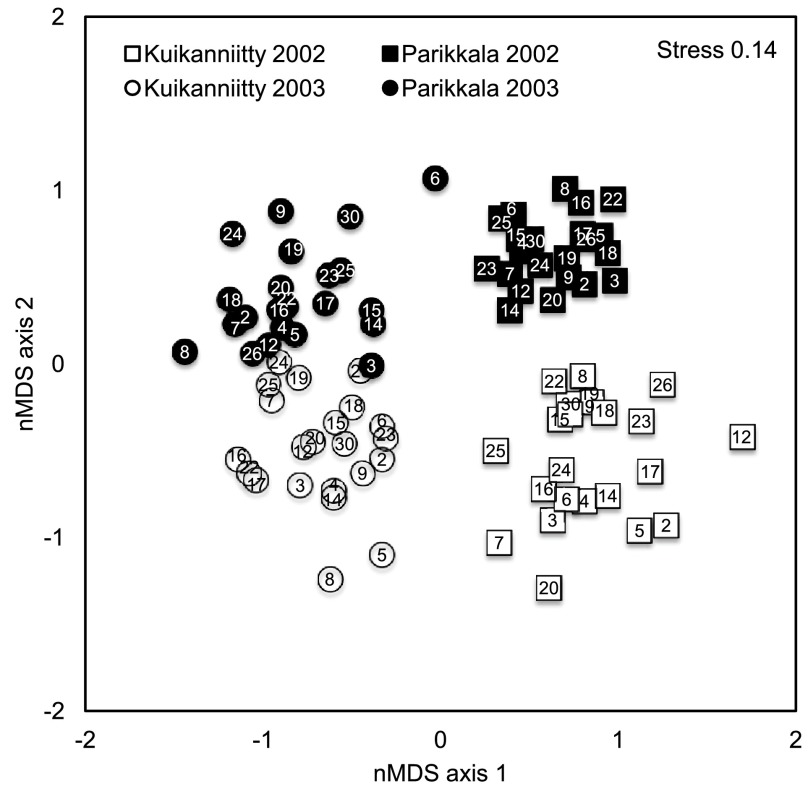
Non-metric MDS plot of insect assemblages of 23 species colonizing 22 *B. pendula* genotypes in Kuikanniitty and Parikkala 2002 and 2003. Stress is 0.14, which indicates a good representation of the data in two-dimensional ordination plot. Each point is a centroid of six replicates. Numbers in the centre of the markers are genotype identification numbers.

Study years and sites were distinctly grouped apart into two-dimensional ordination space, when the genotype centroids of different years and sites were analyzed using nMDS ( Figure 1). The MANOVA Table in turn, shows that sites had statistically significantly different insect species community composition in both years ( Table 2). Sites were also clearly different in their total mean abundance ( Table 3) and species richness (p < 0.008 for the site effect in species richness): the forest site of Parikkala had a 49–78% higher total mean abundance, but 18–25% lower species richness than the abandoned field site of Kuikanniitty in 2002–2003, respectively. These findings indicate that each year and site had significantly different herbivorous insect assemblages, thus creating different biotic environments.

**Table 2.** Non-parametric MANOVA table of the effects of genotype and site on insect herbivore community structure on *B. pendula* saplings in 2002–2003. Sapling size index, which is a measure of height and number of leaves (see material and methods), was used as a covariate.

	*Insect herbivore community 2002*				*Insect herbivore community 2003*			
	*df*	SS	F	P	*df*	SS	F	P
Genotype	21	27700	1.26	0.04	21	33294	2.04	< 0.001
Site	1	47798	16.99	< 0.001	1	10319	7.62	< 0.001
Block (Site)	10	17826	1.70	0.002	10	18013	2.31	< 0.001
G × S	21	22242	1.01	0.466	21	22472	1.37	0.006
Size index	1	4890	4.65	< 0.001	1	8084	10.38	< 0.001
Residual	208	218510			208	162000		
Total	262	344260			262	274880		

**Table 3.** The ANCOVA table of the effects of genotype, block and site on total mean abundance of herbivores (log[x+1] transformed) of *B. pendula* saplings in 2002–2003. Sapling size index, which is a measure of height and number of leaves (see material and methods), was used as a covariate.

	*Mean abundance 2002*				*Mean abundance 2003*			
	*df*	SS	F	p	*df*	SS	F	p
Genotype	21	1.10	2.82	0.011	21	0.26	1.08	0.435
Error	21	0.39			21	0.24		
Site	1	1.13	14.6	0.004	1	0.59	46.7	< 0.001
Error	9.6	0.75			26.7	0.34		
G × S	21	0.39	1.00	0.470	21	0.24	1.81	0.019
Error	208	3.88			208	1.30		
Block (Site)	10	0.77	4.11	< 0.001	10	0.16	2.55	0.006
Error	208	3.88			208	1.30		
Size index	1	0.30	16.3	< 0.001	1	0.23	36.4	< 0.001
Error	208	3.88			208	1.30		

### Genotypic variation and genotype × environment interactions

**Figure 2.**
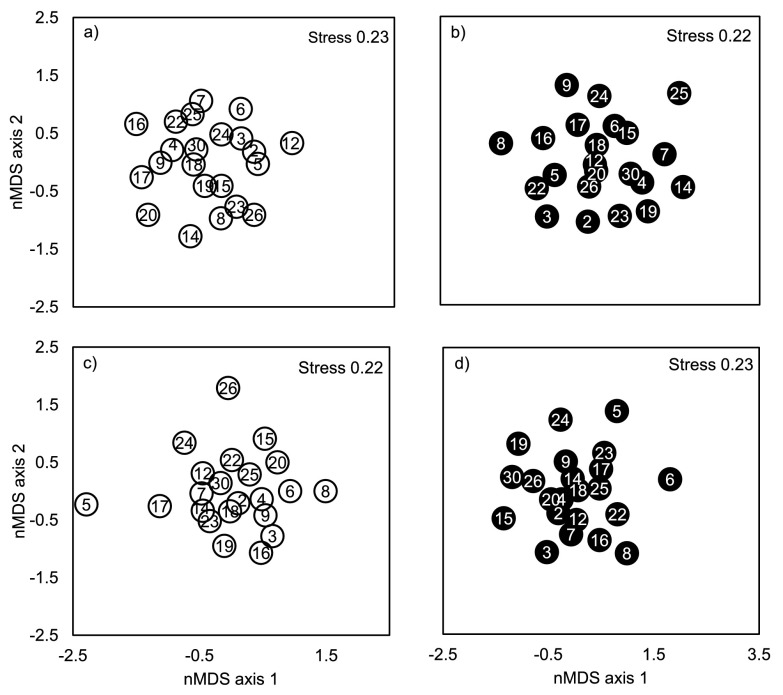
Non-metric MDS plot of insect assemblages of 23 (Parikkala 2002) or 27 species colonizing *B. pendula* genotypes in **a**) Kuikanniitty 2002, **b**) Parikkala 2002, **c**) Kuikanniitty 2003 and **d**) Parikkala 2003. Each point is a centroid of six replicates. Numbers in the centre of the markers are genotype identification numbers. White circles denote genotypes in Kuikanniitty, black circles denote genotypes in Parikkala. Stress values >0.2 indicate that this data may be better visualized with more dimensions (stress for three-dimensional solutions varied between 0.14 to 0.16).

*B. pendula* genotypes were significantly different in their insect species community composition in both study years ( Table 2). In 2002, regional scale environmental (site) variation explained more of the total variation in species composition than the genotype (13.9 and 8.0%, respectively), while in 2003 the genotype explained more of the total variation than the site (12.1 and 3.8%, respectively). Significant genotype × site interaction, which explained 8.2% of the total variation, was found only in 2003. When the sites were tested separately in both years we found that the effect of genotype was significant in Kuikanniitty 2002 and both study sites in 2003 ( Table 4, Figure 2). *B. pendula* genotype could account for 15.8–27.0% of the total variation in community structure, while local scale environmental (block) variation explained 5.9–7.6% of the total variation in community structure ( Table 4, Figure 2).

**Table 4.** Non-parametric MANOVA table of the effects of genotype and block on insect herbivore community structure on *B. pendula* saplings in Kuikanniitty and Parikkala 2002–2003. Sapling size index, which is a measure of height and number of leaves (see material and methods), was used as a covariate.

	*Insect herbivore community 2002*				*Insect herbivore community 2003*			
	*df*	SS	F	P	*df*	SS	F	P
*Kuikanniitty*								
Genotype	21	33778	1.29	0.018	21	25678	1.33	0.013
Block	5	10803	1.73	0.006	5	10305	2.25	< 0.001
Size index	1	4468	3.58	0.001	1	5948	6.48	< 0.001
Residual	103	128690			103	94495		
Total	130	181760			130	138930		
*Parikkala*								
Genotype	21	16771	1.08	0.297	21	29083	2.16	< 0.001
Block	5	8067	2.17	< 0.001	5	7754	2.41	< 0.001
Size index	1	1735	2.34	0.033	1	2811	4.38	0.001
Residual	104	77278			104	66834		
Total	131	105910			131	107680		

**Figure 3.**
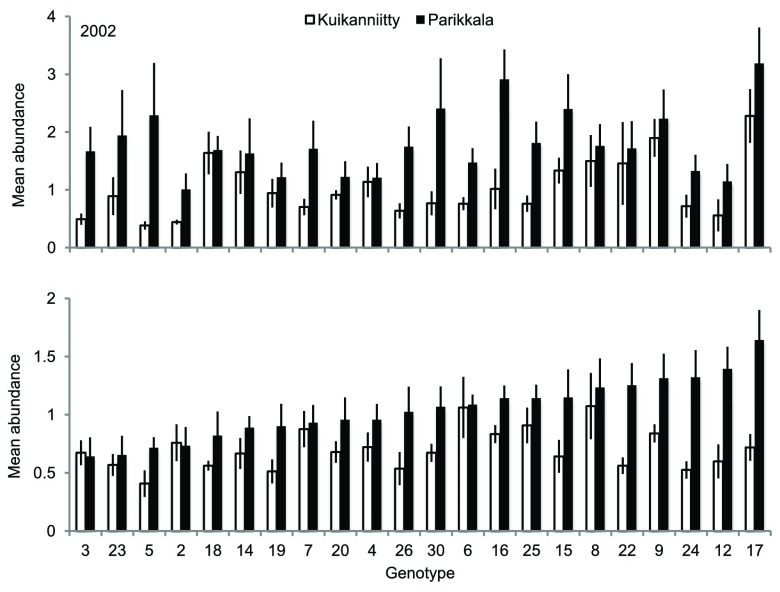
Mean abundance of insect herbivores (±SE) among *B. pendula* genotypes in Kuikanniitty and Parikkala study sites in 2002–2003. White bars: Kuikanniitty, black bars: Parikkala.

*B. pendula* genotypes also significantly differed in their total mean abundance of herbivores (mean number of herbivores/sapling): the total mean abundance of the most susceptible genotype was 5.4- and 3.2-fold compared to the total mean abundance of the most resistant genotype in Kuikanniitty and Parikkala 2002, respectively ( Table 3, Figure 3). In 2003, only the genotype × site interaction was statistically significant, which indicates that the genotype effect strongly depended on the study site. Indeed, when we tested the study sites separately, genotype effect was significant only in Parikkala (ANCOVA: Parikkala F _21,104_=2.29, p=0.003; Kuikanniitty F _21,103_=1.48, p=0.103). The species richness (number of insect species/sapling) was not significantly affected by the *B. pendula* genotype or genotype × site interactions in either year (p>0.134).

Local scale genotype × environment interaction (i.e. the interaction of genotype × replicated block) was studied in Kuikanniitty 2003. Insect species community composition was significantly affected by both genotype and genotype × block interaction ( Table 5). Genotype variation explained 10.6% and genotype × block variation 38.0% of the total variation in insect community composition, indicating that genotype effect is also strongly affected by local scale environmental variation. Total mean abundance or species richness was not affected by genotype or genotype × block interaction (p>0.097).

### Effects of the previous year’s herbivory on insect communities

**Figure 4.**
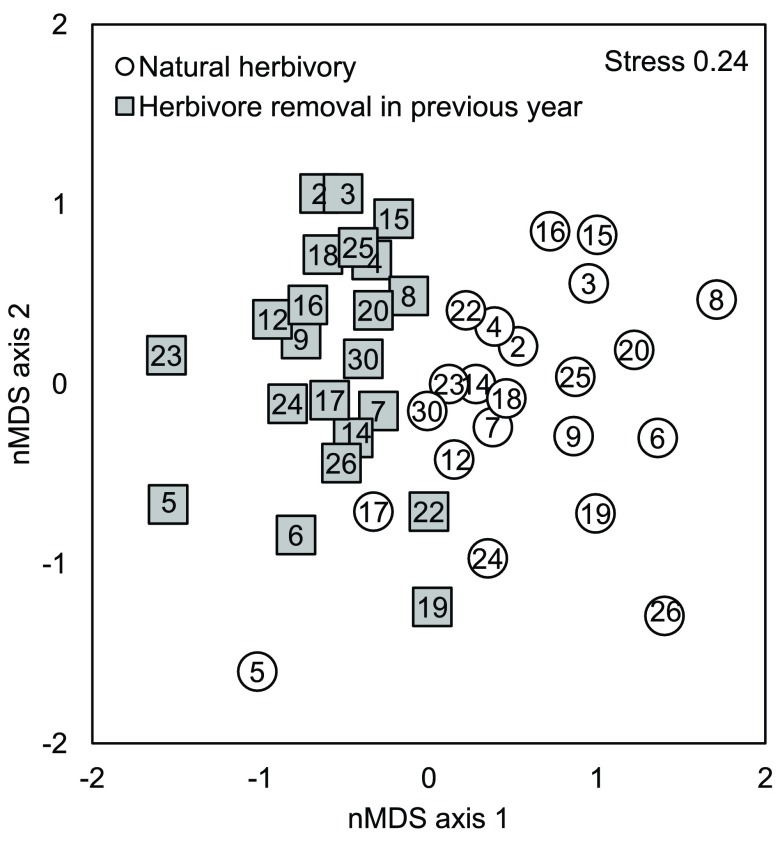
Non-metric MDS plots of insect assemblages of 27 species colonizing 22 *B. pendula* genotypes that were either subjected to natural herbivory or protected from herbivory in the previous growing season in Kuikanniitty 2003. Each point is a centroid of six replicates. Numbers in the centre of the markers are genotype identification numbers.

Previous year herbivory changed the insect community composition of *B. pendula* saplings ( Table 5). The genotype centroids of those saplings that were either subjected to natural herbivory or protected from it were located on the opposite sides of the two-dimensional nMDS ordination plot, although overlapping is evident ( Figure 4). Previous year herbivory did, however, explain only 4.4% of the total variation in insect community composition. Total mean abundance was affected by the previous year’s herbivory as well, but species richness was not (ANCOVA: effects of insect removal on total mean abundance F _1,5.28_=34.6, p=0.002 and species richness p>0.829).

**Table 5.** Non-parametric MANOVA table of the effects of genotype, block and previous year insect removal on insect herbivore community structure among *B. pendula* saplings in Kuikanniitty 2003. Sapling size index, which is a measure of height and number of leaves (see material and methods), was used as a covariate.

	*df*	SS	F	P
Genotype	21	30358	1.39	0.004
Insect removal	1	12525	7.11	< 0.001
Block	5	10930	2.13	< 0.001
G × IR	21	15557	0.90	0.743
G × B	104	109030	1.27	0.002
IR × B	5	6031	1.46	0.069
Size index	1	2078	2.52	0.023
Residual	103	84964		
Total	261	286640		

### Correlations between species

The associations between insect species across genotypes in different sites and years seemed to be based on random associations, since we found only one correlation that was significant after sequential Bonferroni correction ^40^. An unidentified gallery mine (Lepidoptera 4) and *E. fuliginosella* were correlated across genotypes in Kuikanniitty 2002 (Pearson’s correlation; in 2002, r = 0.88, n = 22, p < 0.0001; in 2003, r = 0.545, n = 22, p = 0.009), but not in Parikkala (p>0.199).

Community structure of insect herbivores on different genotypes of silver birch (Betula pendula) 2002_Data: Abundances of 23 individual insect species on 22 birch genotypes planted in 6 blocks in both Kuikanniitty and Parikkala study sites in 2002. Data also contain the columns for species richness and total mean abundance. Those four species that were not surveyed in Parikkala 2002 were excluded from Kuikanniitty species richness and total mean abundance calculations to better enable site comparisons. Columns containing species counts are named as in Table 1 in the associated article. Site: 1=Kuikanniitty, 2=Parikkala
2003_Data: Abundances of 27 individual insect species on 22 birch genotypes planted in 6 blocks in both Kuikanniitty and Parikkala study sites in 2003. Data also contain the columns for species richness and total mean abundance. Columns containing species counts are named as in Table 1 in the associated article. Site: 1=Kuikanniitty, 2=Parikkala.
2003_Additional_KuikanniittyData: Abundances of 27 individual insect species on 22 birch genotypes planted in 6 blocks in Kuikanniitty. Each block has two saplings from each genotype: one that was sprayed with pyrethrin in 2002 and one non-sprayed control. Data also contain the columns for species richness and total mean abundance. Columns containing species counts are named as in Table 1 in the associated article. Insecticide: 0=control, i.e. natural herbivory, 1=herbivores removed by pyrethrin sprayings in 2002. Site: 1=Kuikanniitty.

## Discussion

Our results provide evidence that genetic variation within a natural *B. pendula* population can modify the structure of the arthropod community even though all genotypes supported similar insect species richness. Genetic variation in phenotypic plasticity, however, seemed to be the major factor affecting the abundance and structure of the insect herbivores associated with this tree species, because genotype effect was often dependent on the environmental variation at both regional ( Table 2 and Table 3) and local scales ( Table 5). Those *B. pendula* genotypes that were used in our study should give unbiased estimates of the true variance that is present in *B. pendula* populations, since we chose them randomly from one naturally regenerated population stand (< 0.9 ha) in eastern Finland, where this Eurasian deciduous tree species is particularly abundant ^42^. By contrast, we might have exaggerated the role of regional environmental variation and genotype × environment (site) interactions by planting our genotypes on two rather different areas (open forest and abandoned field, areas that are typically rapidly colonized by *B. pendula*) at a much larger scale (70,000 ha). Therefore, it is not surprising that the importance of the genetic variation in structuring insect herbivore communities of *B. pendula* decreased from 15.8–27.0% (of variation explained) to 8.0–12.1% with increasing spatial scale in our study. Other studies have also found that while the effect of a genotype can be clear on local scales (within common gardens), it may be partially swamped by environmental variation on larger scales ^7, 43^.

It has been argued that, because host plant genotypes have often been collected from large geographic areas and studied within the confines of a single common garden, the role of the host plant genotype in arthropod community patterns has been largely overestimated ^12^. Indeed, Tack *et al.* ^13^ showed that spatial processes dominated genetic effects when genotypes of *Q. robur* were collected at the same local (500 ha) or regional (1 million ha) scale as that where experiments were conducted, and thus, in real landscapes, spatial impacts might relegate host plant genotype to a minor role. Our results, however, suggest otherwise, because ** genotype explained about three times more of the total variation in insect herbivore community structure than local environment (block) in both sites ( Table 4), and the scale of our common garden(s) was approximately the same as the scale of that where genotypes were collected (< 0.9 ha). In addition, on a regional scale, genetic and environmental effects explained similar proportions of the total variation in arthropod community structure ( Table 2), even though we might have inflated the role of the environment in our study. This discrepancy in our results might perhaps be attributed to the difference in the distribution of these wind-pollinated tree species: the populations of *Q. robur* are strongly fragmented and grow at the northern margin of the species’ European distribution in southern Finland (where Tack *et al.* ^13^ conducted their experiments), while *B. pendula* has a wider and more continuous distribution over the whole of Finland, apart from Lapland. *Q. robur* populations exhibit higher geographic differentiation estimates, *F*_st_ 0.032 for *B. pendula* and 0.066 for *Q. robur* ^44, 45^, which means that the gene flow among *B. pendula* populations is two times higher than among *Q. robur* populations, and thus local *B. pendula* populations might express a larger amount of genetic variation than populations of *Q. robur*.

We found that insect herbivore communities can be affected by both local and regional genotype × environment interactions, at least in some years. But why do *B. pendula* genotypes support different insect communities in different environments? It is possible that resistance traits of the genotypes are changed due to differences in abiotic environment and insect communities respond to these changes. This is supported by the fact that earlier studies have found regional genotype × environment interactions in the secondary metabolites of the same study saplings ^26^. Yet, we do not know whether genotype × environment interactions in *B. pendula* resistance traits exist at a local scale and recent studies suggest that secondary metabolites are not the most important anti-herbivore defence of plants ^31^. On the other hand, spatial processes might affect local insect communities and create genotype × environment interactions. For example, in our experiment where genotypes of each block are arranged randomly, the effects of a particular genotype could be partially masked by the effects of their conspecifics in some blocks if nearby genotypes are very dissimilar, i.e. there is associational resistance (see a review by Agrawal *et al.* ^46^) at the level of a genotype. Both of these processes may be affecting different insect species differently. We found only one species pair that was correlated across genotypes in one of our study sites, which, together with earlier findings ^47, 48^, indicates that generalized defenses against multiple insect species are not likely in *B. pendula* (see Leimu and Koricheva ^49^). Additionally, it may also be that local insect communities differ in their response regardless of spatial processes and without any change in the traits of *B. pendula*.

The size of *B. pendula* trees is positively associated with their fitness, i.e. seed production ^29^. It has been shown that herbivores can reduce the growth of *B. pendula* by up to 46% (Mikola *et al.* unpublished results, see also Prittinen *et al.* ^22^, Silfver *et al.* ^23^) and increase seedling mortality considerably ^50^. Thus, by imposing selection in various genetically variable resistance traits of *B. pendula* ^25, 26, 51^, herbivores may have high potential to drive the community evolution in *B. pendula.* Indeed, we found that only one season of protection from herbivory changed arthropod community variables (mean abundance and community composition) in five-year old field-grown *B. pendula* saplings. Total mean abundance, for example, was lower in saplings that were protected from herbivory in the previous growing season, which indicates that they may have had more resources to defend themselves against insects when herbivores were present again. Yet, the magnitude of these effects was smaller than the effects of local environmental (block) variation, and could explain only about 4% of the total variation in arthropod community structure. It is important to note, however, that in nature *B. pendula* seedlings typically establish in open patches, where high numbers of individuals compete heavily before self-thinning eliminates some of the seedlings. Surviving for these first years and consequently reaching maturity is crucial for an individual’s fitness in this long-lived tree species. Earlier studies that have used open-pollinated progeny of the same genotypes, have shown that in such dense stands, even moderate levels of insect herbivory can change the genetic structure of *B. pendula* populations in the first year of establishment ^52^. This is reminiscent of recent studies, which have demonstrated that natural selection can favour different genotypes in the absence of herbivores rather than in their presence, and different genotypes in response to different herbivore species within only few generations of annual or biannual plants ^53, 54^ (see also Hare ^55^).

To conclude, we have shown that the structure of insect herbivore communities can be significantly affected by intraspecific genetic variation when there is no mismatch in scale. However, genetic effects were modified by environmental variation on both a local and regional scale in one study year. Furthermore, insect herbivore damage in one growing season changed the community patterns of the following season, yet those effects were minimal compared to genetic and environmental factors. Our results suggest that both genetic and environmental factors are important determinants of the community structure of herbivorous insects. Together these mechanisms appear to maintain the high diversity of insects in *B. pendula* forest ecosystems.

## Data availability

figshare: Community structure of insect herbivores on different genotypes of silver birch ( *Betula pendula*), http://dx.doi.org/10.6084/m9.figshare.915332 ^56^
