# Supplementary material for: Genetic and environmental determinants of insect herbivore community structure in a Betula pendula population
Source: F1000Res. 2014 Jan 31;3:34. [Version 1] doi: 10.12688/f1000research.3-34.v1 (PMC3962004; doi:10.12688/f1000research.3-34.v1)
Supplement: Peer review history

# Peer review: Referee response for version 1

**Published:** 2014-02-24

**Authors:** Judith Myers

**Affiliations:** 1 Department of Zoology, University of of British Columbia, Vancouver, BC, Canada

This is an interesting study on the role of tree genotypes on herbivorous insect attack of willow trees, *Betula pendula,* using a common garden approach in Finland. Twenty-two trees were randomly selected and cloned, although no details are given as to how the trees were cloned. Each tree is considered to be a different genotype. Six blocks with 4 saplings of each genotype were used from which one sapling was randomly selected to give 6 replicates per genotype for the study of insect attack. Insect sampling was done largely based on the damage done over the summer. I am surprised that it is possible to distinguish insects based on their damage as I would have guessed that the damage from leaf miners would have been similar, and damage from sucking insects difficult to find at all. I have the following questions and comments:

1. What is meant by structure of the insect community? This term is used in the abstract and throughout the paper but it is not defined.
   
2. The term local environmental variation is mentioned, and it would be clearer if this was referred to as variation among blocks.
   
3. Insects were removed from some saplings but details are lacking on how this was done, how frequently it was done, and how effective it was.
   
4. “Additional Kuikanniitty 2003 data combined with Kuikanniitty 2003 raw data was used” I don’t understand what this means.
   
5. How would these results be interpreted by one who wanted to select for herbivore resistance among tree genotypes? Is the amount and consistency of the among genotype resistance sufficient to evolve over time?
   
6. How are the results influenced by variation in insect abundance from year to year? 

I have read this submission. I believe that I have an appropriate level of expertise to confirm that it is of an acceptable scientific standard.

# Peer review: Referee response for version 1

**Published:** 2014-02-07

**Authors:** Patrick Tobin

**Affiliations:** 1 Ecology and Management of Invasive Species and Forest Ecosystems Unit, United States Department of Agriculture, Morgantown, WV, USA

In this study, the authors selected and cloned 22 silver birch genotypes and planted them at two common garden sites to examine the effects of both genetic and environmental factors on the community structure of insect herbivores. By selecting genotypes from a spatially limited area (< 9 ha), and by performing the experiments at two spatially separated common garden sites, the authors were able to examine more precisely the main and interacting effects of genetic and environmental variation on the herbivore community. The authors also excluded herbivores on half of the host trees at each common garden site through application of an insecticide, which allowed them to examine the effect on the herbivore community in the following year.

 

One limitation of the study is the use of only two common garden sites, which does limit the broader implications of the study. However, despite these limitations, the data still provide sound preliminary information on the importance of both genetic and environmental variation on the structure of the herbivore community.

 

Although I understand and appreciate the challenges of documenting the herbivore community and the labor involved to do so, more comments are needed to address the lack of a more temporally robust sampling regime. Some species were sampled at a specific time given their respective seasonality, but most were sampled at the end of the growing season in the fall. In doing so, I suspect that authors would have missed spring and summer feeders, such as the winter moth for example, whose damage could have been difficult to ascertain and differentiate from other herbivores when herbivore damage was examined in the fall. This is not a fatal flaw, especially since the authors incorporated herbivore exclusions, which presumably would have also excluded spring and summer feeding herbivores. However, since one goal of the study was to examine the role of the herbivore community in affecting the community in the following year, it would be helpful to discuss the potential limitations of the fall sampling regime in documenting insects that feed earlier, and how the herbivore exclusion component was also (presumably) a mechanism to deal with sampling limitations. On a related note, it wasn't clear to me if potential temporal autocorrelation in the numbers and diversity of the herbivore community from one year to the next was appropriately addressed in the analysis; if so, I would suggest adding a statement in the materials and methods how this was addressed or if not, why it did not need to be addressed.

 

Overall, this was a nicely designed experiment, the manuscript was very well written, and the data were well presented. This study adds to our knowledge of the role of genetic and environmental variation on the structure of the herbivore community on silver birch, and sets the stage for a number of interesting follow-up questions.

I have read this submission. I believe that I have an appropriate level of expertise to confirm that it is of an acceptable scientific standard.
